# Immunotherapy in Solid Tumors and Gut Microbiota: The Correlation—A Special Reference to Colorectal Cancer

**DOI:** 10.3390/cancers13010043

**Published:** 2020-12-25

**Authors:** Asimina Koulouridi, Ippokratis Messaritakis, Nikolaos Gouvas, John Tsiaoussis, John Souglakos

**Affiliations:** 1 Laboratory of Translational Oncology, School of Medicine, University of Crete, 70013 Heraklion, Greece; asi_minakoulouridi@yahoo.com; 2 Medical School, University of Cyprus, 20537 Nicosia, Cyprus; nikos.gouvas@gmail.com; 3 Department of Anatomy, School of Medicine, University of Crete, 70013 Heraklion, Greece; tsiaoussis@uoc.gr; 4 Department of Medical Oncology, University Hospital of Heraklion, 71110 Heraklion, Greece

**Keywords:** immunotherapy, immune checkpoint inhibitors, gut microbiota, colorectal cancer

## Abstract

**Simple Summary:**

Immunotherapy and immune checkpoint inhibitors have become the breakthrough treatment with extended responses and survival rates in various neoplasms. They use the immune system to defeat cancer, while gut microbiota seems to play a significant role in that attempt. To date, colorectal cancer patients have gained little benefit from immunotherapy. Only mismatch repair-deficient/microsatellite-unstable tumors seem to respond positively to immunotherapy. However, gut microbiota could be the key to expanding the use of immunotherapy to a greater range of colorectal cancer patients. In the current review study, the authors aimed to present and analyze the mechanisms of action and resistance of immunotherapy and the types of immune checkpoint inhibitors (ICIs) as well as their correlation to gut microbiota. A special reference will be made in the association of immunotherapy and gut microbiota in the colorectal cancer setting.

**Abstract:**

Over the last few years, immunotherapy has been considered as a key player in the treatment of solid tumors. Immune checkpoint inhibitors (ICIs) have become the breakthrough treatment, with prolonged responses and improved survival results. ICIs use the immune system to defeat cancer by breaking the axes that allow tumors to escape immune surveillance. Innate and adaptive immunity are involved in mechanisms against tumor growth. The gut microbiome and its role in such mechanisms is a relatively new study field. The presence of a high microbial variation in the gut seems to be remarkably important for the efficacy of immunotherapy, interfering with innate immunity. Metabolic and immunity pathways are related with specific gut microbiota composition. Various studies have explored the composition of gut microbiota in correlation with the effectiveness of immunotherapy. Colorectal cancer (CRC) patients have gained little benefit from immunotherapy until now. Only mismatch repair-deficient/microsatellite-unstable tumors seem to respond positively to immunotherapy. However, gut microbiota could be the key to expanding the use of immunotherapy to a greater range of CRC patients.

## 1. Introduction

There is no doubt that in recent years, a breakthrough has been achieved by introducing immunotherapy as a treatment option for solid tumors. In the past, cytotoxic agents were the main choice for cancer therapy. It was not earlier than 1980 that W. Colley demonstrated that the inoculation of *Streptococcus pyogenes* bacteria in sarcoma patients led to cancer remission. This proof-of-concept study demonstrated that the immune system and its modification could be a potential tool against cancer [1]. The immune system uses two major pathways to defeat an intruder. First, immune cells recognize the pathogen and release of a number of different cytokines, and activation of complement and phagocytosis follow. Then, the adaptive immune system is activated, using B and T lymphocytes for the elimination of the pathogen [2]. The understanding of such mechanisms and their correlation with cancer elimination has led to many innovations and new treatment options, like immune checkpoint inhibitors (ICIs).

The human body is colonized by more than 100 trillion microbes that include about 4930 bacterial, eukaryotic and archaeal species, most of which are in the gut. Gut microbiota colonization starts at the time of birth and is remodeled according to diet, way of living, diseases, aging, drug consumption and other environmental factors. The gut of a healthy individual is mainly composed of microbes of *Firmicutes* and *Bacteroidetes* phyla, whereas *Cyanobacteria*, *Proteobacteria*, *Actinobacteria*, *Fusobacteria* and *Verrucomicrobia* phyla appear in a minor proportion [3], which interact with the host directly or through their products, thus regulating homeostasis, inflammation, metabolism and immunity [4,5]. Dysbiosis and microbial translocation have been associated with many health disorders, including inflammations, autoimmune disorders and precancerous or cancerous lesions [6]. It has already been demonstrated that gut microbiota, as an immunomodulator and a modulator of metabolism, could affect the efficacy of immunotherapy in cancer patients [7,8,9,10,11,12,13,14,15,16,17].

Colorectal cancer (CRC) is the third most common cancer worldwide, and in the USA alone, the disease is estimated to cost the medical care system $17 billion in 2020 [18]. Although significant improvements have been made towards the cure of CRC, immunotherapy has a minor role. To date, only patients with mismatch repair-deficient (dMMR) or microsatellite instability-high (MSH) tumors seem to benefit from immunotherapy, thus leading to FDA approval for ICIs only for dMMR/MSH CRC patients [19,20]. However, only about 15% of all CRCs are dMMR/MSH [21,22]. Under these circumstances, new ways are under evaluation to maximize the use and efficacy of immunotherapy in CRC patients. Gut microbiota seems to have a key role in this procedure, and extensive research has been done about this hypothesis.

In this review study, we aimed to present and analyze the mechanisms of action and resistance of immunotherapy and the types of ICIs, as well as their correlation to gut microbiota. A special reference will be made in the association of immunotherapy and gut microbiota in the CRC setting.

## 2. Immunotherapy in Solid Tumors

Immunotherapy is a relatively new treatment option and has become a powerful clinical strategy against solid tumors. However, not all tumors react in the same manner (Table 1). So far, there are two major ICIs classes: the cytotoxic T-lymphocyte associated protein 4 (CTLA-4) and the programmed death receptor and its ligand 1 (PD-1/PD-L1) inhibitors. CTLA-4 is overexpressed on regulatory T lymphocytes (Tregs) and activated CD4+ and CD8+ T cells, whereas, anti-CTLA-4 antibodies enhance anti-tumor immunity [23,24]. PD-L1 is expressed by stromal and tumor cells. PD-L1 binds to its receptor (PD-1) on cytotoxic T-lymphocytes (CTLs). Anti-PD-L1 and anti-PD-1 immunotherapy target this complex and the immunosuppression that is provoked [25]. The approved ICIs include a. anti-CTLA-4: ipilimumab; b. anti-PD-L1: nivolumab, durvalumab, avelumab; and c. anti-PD-1: atezolizumab and pembrolizumab. ICIs are used for a great number of solid tumors, including melanoma, renal cell cancer, hepatocellular cancer, urothelial carcinoma, head and neck carcinoma, non-small and small cell lung cancer, dMMR/MSH CRC or non-CRC and gastric cancer [24].

ICIs’ modes of action include the pattern recognition receptors (PRRs), expressed by macrophages and dendritic cells [27]. Foreign molecules and endogenous stress signals notify the body of the presence of an invader or damage. Then, immune cells release chemokines and type I interferon, leading to further inflammation and CTLs infiltration. Moreover, dendritic cells present the tumor neo-antigens to CTLs [24]. Following these mechanisms, ICIs either eliminate the activation of Tregs or provoke dendritic cells to release IL-12, leading to further activation of CTLs, thus fighting tumor cells [25].

However, immunotherapy seems to be inefficient against every tumor. The question is how this resistance is achieved. It seems to be a complex phenomenon, and two main mechanisms have been described: The first mechanism includes the tumor microenvironment, which overcomes the immune surveillance using its components [28]. It consists of various immunosuppressive cells, such as Tregs, which express a high amount of fork-head box P3 (FoxP3) transcription factor, secrete inhibitory cytokines and suppress effector T lymphocytes (Teff) [29,30]. B regulatory cells (B-regs) suppress cytotoxic CD4+ and CD8+ Teffs [30]. Myeloid-derived suppressor cells (MDSCs), by the expression of molecules like CD11b and Gr-1, promote immunosuppression against cancer and cancer invasion [31]. M1 tumor-associated macrophages (TAMs) seem to be implicated in the tumor immunosurveillance and M2 in tumorigenesis [32,33]. Other components of the stroma are the tumor-associated mast cells (TAMCs) which promote angiogenesis, tumorigenesis and immunosuppression by releasing a number of proteolytic enzymes and growth factors [34]. The second mechanism that affects the efficacy of immunotherapy is autophagy. Autophagy regulates the tumor microenvironment, making metabolic changes, creating hypoxic conditions and producing inflammatory and immunosuppressive components [35]. Autophagy plays an important role in the antigen presentation on the surface of antigen-presenting cells and as a regulator of the major histocompatibility complex [28,36].

Resistance to immunotherapy could also be triggered by molecular changes, oncogenes, genetic or epigenetic alterations and the number of neo-antigens, which are directly related to tumor mutational burden [28,37].

## 3. Gut Microbiota and Immunotherapy

Gut microbiota seems to be another regulator of immunotherapy response. Studies have shown that the diversity and abundance of gut microbiota could determine response to immunotherapy by various pathways [6,38,39]. Gut microbiota produces metabolites and forms biofilms that regulate metabolism, inflammation and immunity [38]. Over the last few years, the development of technology and algorithms allowed the easier and more effective detection and identification of these microorganisms and the testing of their collective contribution to human health without the need for their isolation and culture. Such technology includes next-generation sequencing of the 16S ribosomal RNA and, more recently, whole-genome shotgun sequencing and metagenomic sequencing [40].

### 3.1. Interference of Gut Microbiota and Immunity

Recent studies have demonstrated that gut microbiota affect not only the efficacy of immunotherapy but also the frequency and intensity of its adverse events [12,24]. Toll-like (TLRs) and NOD-like receptors (NLRs) are the two major PRRs that are implicated in immunity [41,42]. There are nine TLRs subtypes: TLR1,2 and 4–6 recognize the extracellular bacterial components expressed on the cell surface, whereas TLR3 and 7–9 recognize primarily viral nucleic acids [43]. TLRs bind to their ligands and are involved in signal transduction through the Toll/interleukin-1 receptor (TIR)-domain containing adapter-inducing interferon-β (TRIF) and myeloid differentiation of primary response protein 88 (MYD88). TLR uses either MYD88 or TRIF to transfer the signal, usually by creating heterodimers with other TLRs or complexes with specific adapters. All these steps lead to the activation of nuclear factor-kappa B (NF-κΒ) pathway and the inflammation response [42,44,45]. Deletion of MYD88 signal in the epithelium leads to a numerical increase of mucus-associated microorganisms and their translocation to mesenteric lymph nodes [46], which is correlated with the development and progression of some cancer types, such as CRC, and their clinical outcome [6].

NLRs are a big family of receptors that includes: nucleotide-binding oligomerization domain-1 (NODs), NACHT-, Leucine-rich repeat- and pyrin- domain-containing proteins, Interleukin 1β converting enzyme-protease activating factor, neuronal apoptosis inhibitor factors and class II major histocompatibility complex transactivator [47]. Each NLR has a unique ligand, recognizing specific microbial components or products and participates in a different manner to the action of innate immunity. For example, *Listeria monocytogenes* toxin is the activator of NLR6. NLR6 combines with the adapter apoptosis-associated speck-like protein containing a C-terminal caspase recruitment domain, forming inflammasomes in the cytoplasm and leading to the recruitment of caspase-1 and the release of IL-1β, thus shutting down inflammatory response [48,49]. All mechanisms that include NLRs seem to play a critical role in the activation of the immune system in the intestinal epithelium, tumor monitoring and immunosurveillance [50].

Concerning adaptive immunity, pathogen-associated molecular patterns activate the antigen-presenting cells (APCs), which migrate to mesenteric lymph nodes, where naïve T lymphocytes mature into CD4+ T cells. These cells are differentiated into T helper (Th) cells and Tregs [42,51]. These two subtypes are crucial for maintaining a symbiotic balance between the immune system and microbiota, affecting tumor growth and immunosurveillance. Th cells act as regulators by selecting a specific immunoglobin A (IgA) plasma cell bank, expressing PD-1, which binds to PD-L1 on the surface of B cells [52,53]. PD-1-deficient mice produce IgAs not capable to properly bind bacteria, and bacterial diversity is disturbed in companion with antibodies required to maintain a mucosal barrier, thus leading to dysbiosis [54]. Th17 cells play an important role in tumorigenesis through the secretion of IL-17f. There are conflicting data on different cancer types and the role of IL-17f. For instance, in non-small cell lung cancer, IL-17f increases the invasion by promoting angiogenetic chemokines. On the contrary, in CRC, IL-17f suspends angiogenesis through the reduction of vascular endothelial growth factor [55,56].

Tregs express PD-1 and PD-L1 and are in abundance in the lamina propria of the colon. The PD-1/PD-L1 axis also seems to be one of the inhibitors of CD4+ and CD8+ T lymphocytes response, using specific cytokines like transforming growth factor (TGF)-β and IL-10 [42,57,58]. Not only microorganisms, but also their fractions and metabolites, can alter the immune response and interfere with Tregs regulation. These metabolites, such as short-chain fatty acids, activate pathways that can induce the release of IL-10 [59], increasing the response of T cells to TGF-β1 by regulating the TGF receptor expression [60]. TGF-β, in parallel, increases the expression of Foxp3 in CD25 T cells, converting them to CD4+ CD25+ induced regulatory T cells (iTregs) [57], thus leading to further immunosuppression. Other metabolites that could act as immunomodulators through the regulation of Tregs are the symbiotic factor polysaccharide A and butyrate. The symbiotic factor polysaccharide A is produced by *Bacteroides fragilis*, leading to increased expression of Foxp3, IL-10 and TGF-β through the motivation of TLR2 on CD4+ cells. Butyrate increases Tregs through activation of Foxp3 [42]. Dysbiosis leads to the change of quantity and ratio of metabolites, thus leading to different immune reactions. It has been demonstrated that an increase in *Clostridium* spp. and *B. fragilis* led to an increase in iTregs in the gut [39]. iTregs, through metabolic and proliferating pathways, play an important role in PD-1/PD-L1 axis and in the efficacy of anti-PD-1/PD-L1 immunotherapy; however, the mechanism remains to be explored [61,62].

### 3.2. Correlation of Gut Microbiota and ICIs Efficacy: The Proofs

Many studies have concluded that gut microbiota can interfere with the efficacy of immunotherapy [7,8,9,10,11]. The breakthrough of this scenario came by Sivan et al. [7], who enrolled in their study Black-6 mice from different vendors, Taconic Biosciences (TAC) and Jackson Laboratories (JAX), aiming to study the role of microbiota in melanoma development and progression. TAC mice were shown to suffer from more severe melanoma than JAX mice. When TAC and JAX were housed together before melanoma cell implantation, such differences disappeared. Using fecal suspension of the two mice as feeding, it was demonstrated that JAX gut microbiota provided an anti-tumor effect. Combining fecal suspension of JAX mice with anti-PD-L1 immunotherapy led to an improvement of ICI efficacy by increasing T cell responses and decreasing tumor growth, while the shorter tumor growth was correlated with the presence of *Bifidobacterium* spp. [7]. Derosa et al. collected stool samples from both responders and non-responders to immunotherapy who experienced renal cell carcinoma (RCC), non-small cell lung cancer or urothelial carcinoma. The authors performed fecal microbial transplantation in germ-free or antibiotic-treated mice. Mice transplanted with fecal material from responders presented more immuno-efficacy than the non-responders’, leading to tumor reduction. As was demonstrated, the presence of *Akkermansia muciniphila* in the gut contributed to anti-PD1 efficacy [8,63].

Different studies, focused mainly on melanoma, aimed to examine the differences in gut microbiota between responders and non-responders [9,10,11,12]. Frankel et al. used anti-PD-1 and anti-CTLA-4 as a standalone or in combination to treat melanoma patients. It was shown that responders’ gut microbiota was mainly composed of *Faecalibacterium prausnitzii*, *Bacteroides thetaiotamicron*, *Holdemania filiformis* and *Bacteroides caccae* [9]. Gopalakrishnan et al. studied the response and gut microbiota, as well as immunocytes, in melanoma patients under anti-PD-1 treatment. The responders’ (69.8%) gut microbiota was mainly composed of *F. prausnitzii*, *Ruminococcus bromii*, *Porphyromonas pasteri*, *Clostridium hungati* and *Phascolarctobacterium faecium*, whereas gut microbiota of the non-responders was mainly composed of *B. thetaiotaomicron*, *Escherichia coli* and *Anaerotruncus colihomininis* [10]. Immunohistochemistry on tumor specimens and flow cytometry blood samples revealed decreased Tregs and MDSCs with increase in CD8+ T cells and CD68+ HLA-Dr+ CD163+ myeloid dendritic cells [10]. Moreover, Chaput et al. demonstrated correlations of anti-CTLA-4 efficacy and adverse events with gut microbiota in melanoma patients. *F. prausnitzii* L2-6, *Gemmiger formicilitis* ATCC27749, butyrate- producing bacteria SS2-1, *Ruminococcus* spp., *Lachnospiraceae* spp., *Clostridium* XIVa and *Blautia* spp. correlated with better tumor reduction but increased events of ICI-associated colitis. On the contrary, *Bacteroides* spp. were mainly correlated with worse tumor remission and fewer events of colitis [12]. This is in agreement with Dublin et al., who demonstrated ICI-associated colitis in 29.4% of melanoma patients under anti-CTLA-4, and their gut microbiota was mainly composed of microorganisms belonging to the phylum of *Bacteroidetes*, families of *Bacteroidaceae*, *Rikenellaceae* and *Barnesiellaceae* [11]. A recently published meta-analysis of four shotgun metagenomic studies in metastatic melanoma patients receiving immunotherapy showed differences of microbial composition in the gut of responders and non-responders and examined the predictive value of this composition [64]. The gut of responders was enriched with unknown *Ruminococcaceae* and *Faecalibacterium* spp., *Ruminococcus bicirculans* and *Barnesiella intestinihomini*, whereas non-responders were mainly colonized by *B. thetaiotaomicron*, *Adlercreutzia equolifaciens*, *Bifidobacterium dentium* and unknown *Mogibacterium* spp. [64]. Moreover, comparison of gut microbial networks of responders and non-responders revealed that responders’ networks had an abundance of *Mogibacterium*, *Anaerococcus* and *Eggerthella* spp. while non-responders had an abundance of *Subdoligranulum*, *Lachnoclostridium*, *Eggerthella* and *Streptococcus* spp. The abundance of unknown *Faecalibacterium* sp. and of aerobic respiration provided predictive value for the efficacy of immunotherapy [64].

Regarding all the above-mentioned, gut microbiota not only promotes the efficacy of immunotherapy and regulates the emergence of adverse events, but also helps immunotherapy to overcome the tumor’s resistance.

Some microbial species can be synergic to ICIs, while others not. *Bifidobacteria* spp. promote immune response by inducing transcription of dendritic cell genes, leading to their maturation. They can downregulate the threshold of their activation, thus needing less antigens to initiate T cells [7,42]. *B. fragilis* is the activator of Th1 cells, leading them to react with antigens promoting the efficacy of anti-CTLA-4. Moreover, IL-12 is produced as a result of the mobilization of CD11b+ DC to lamina propria by *B. fragilis*, leading to better anti-tumor response [65,66,67]. *A. muciniphila* and *Enterococcus hirae* help to increase central memory tumor cells in the tumor. Such cells have specific chemokine receptors, and the axis in which they are involved leads to better survival rates in some advanced cancers [63]. *Faecalibacterium* spp. promotes dendritic and other APCs in order to achieve CD4+ or CD8+ proliferation and also promotes the production of Tregs, resulting in better anti-CTLA-4 efficacy [26,68]. On the contrary, *Bacteroides* spp. upregulate Tregs and MDSCs, leading to blockage of anti-PD-1 immunotherapy and to an inflammatory response through the TLR-NF pathway that degrades the efficacy of anti-CTLA-4 [69,70].

### 3.3. Gut Microbiota Modulators and Their Impact on ICIs Efficacy

The question that arises is how gut microbiota could be remodeled to obtain the maximum benefit. Apart from fecal microbial transplantation, other exogenous factors are involved in the composition of gut microbiota. The mode of birth delivery and breast or formula feeding during the early life of the infant plays an important role in the jumpstart of gut microbiota. Generally, diet affects gut microbiota. The metabolites produced by the degradation of the food in the gut from different microorganisms play a crucial role in the modulation of different human body’s operations, like immunity [71]. A diet rich in fibers and grains is preferred over one with red meat. Consumption of prebiotics, probiotics or synbiotics can strengthen the microbial function of the gut [72]. Drugs, such as antibiotics or antacids, are also involved in the factors that modulate gut microbiota. Antibiotics have a negative impact on the beneficial bacteria of the gut, decreasing the efficacy of the immunotherapy antitumor treatment [73]. It has been shown that the efficacy of anti-CTLA-4 is decreased in germ-free and specific-pathogen-free mice that received antibiotics in advance [67]. When mice were fed with *B. fragilis* combined with *Burkholderia cepacia* or *B. thetaiotaomicron*, immunotherapy efficacy against tumors was restored through Th1 mediated response and activation of dendritic cells in the tumor [67]. Antibiotics are strongly negatively correlated with overall survival in melanoma, bladder cancer, non-small cell lung cancer and renal cell carcinoma when treated with ICIs [74]. Moreover, the use of corticosteroids has been significantly negatively correlated with ICI efficacy, with an impact on overall survival in melanoma, non-small cell lung cancer and other carcinomas, although there are no adequate prospective data [75].

Additionally, proton-pump inhibitors and metformin have led to the change of gut microbial diversity and taxonomical changes. These changes could have positive or negative effects in tumorigenesis or treatment efficacy [76,77]. Proton-pump inhibitors have been related to worse overall survival and progression free survival in patients with non-small cell lung cancer and urothelial cancer following anti-PD-L1 treatment [78,79]. Case-control and cohort studies present conflicting data about the relation of proton-pump inhibitors use and the risk of CRC [80,81]. However, Lei et al., reported that the extended use of proton-pump inhibitors has been correlated with high risk of CRC [82]. It has been demonstrated that dysbiosis is correlated with decreased ICIs efficacy and increased risk of tumorigenesis [76,83]. The main mechanism is actually the remodeling of microbial diversity, with oral microorganisms, such as *F. nucleatum*, being in high abundance. Proton-pump inhibitors might also lead to bacterial overgrowth in the gut, overproduction of their metabolites and formation of precancerous lesions due to chronic inflammation, as a result of microscopic colitis [76,83,84,85]. On the other hand, metformin, a drug used for the treatment of type 2 diabetes mellitus, targets hepatocytes and the decrease of gluconeogenesis [86]. Studies have concluded that its action can be mediated by the modulation of gut microbiota [86,87,88]. Sun et al., demonstrated that the use of metformin led to the reduction of *B. fragilis* in the gut [86]. Metformin in mice models led to the enrichment of *Verrucomicrobiaceae* and *Prevotellaceae* and depletion of *Lachnospiraceae* and *Rhodobacteraceae* [87]. It has also been shown that in a group of patients with type 2 diabetes mellitus, metformin led to the enrichment of gut microbiota with *Esherichia* and a decrease in *Intestinibacter* spp. [89]. Metformin seems to enhance efficacy of immunotherapy and plays a protective role against tumorigenesis, especially in the case of CRC. Interventions in gut microbiota composition seem to be one of the main mechanisms contributing to these actions [77,90,91]. Metformin enriches gut microbiota with *A. muciniphilla*, a microorganism that has been correlated with better anti-PD-1 efficacy. In addition, metformin increases the short-chain fatty acids-producing bacteria, which could induce Tregs proliferation leading to anti-CTLA 4 sensitivity [77]. Apart from its ability to improve efficacy of immunotherapy, metformin is an antitumor protector, as previously demonstrated [90,92]. Higurashi et al. mention that metformin administration as chemoprevention post polypectomy in non-diabetic patients led to a statistically significantly lower incidence of metachronous adenomas or polyps [92]. Huang et al. showed that metformin can change gut microbiota composition by affecting *Bacteroides*, *Streptococcus*, *Achromobacter*, *Alistipes* and *Fusobacterium* spp. Especially for *F. nucleatum*, which is strongly related to CRC development, metformin relieved the symptoms in APC^min/+^ mice [90,93].

## 4. Immunotherapy and Microbiota in CRC

CRC is one of the leading causes of death due to cancer worldwide. It is a matter of public health, as it financially burdens different health systems, and its early diagnosis by a colonoscopy or a stool-based test for the detection of hemoglobin can have a great impact on the survival of patients [94]. Immunotherapy is a novelty for the last few years for the treatment of different types of cancer. However, in the case of CRC, only a subgroup of CRCs can benefit from immunotherapy. In particular, only anti-PD-1 has approval for its use in metastatic dMMR/MSH CRC [20,95]. It is challenging to understand how to improve the efficacy of immunotherapy not only in dMMR/MSH CRC but also in MMR-proficient CRC. The rationale behind the use of immunotherapy in CRC was that especially dMMR/MSH tumors have a higher amount of neo-antigens capable to enforce immunity against tumors [14].

Gut microbiota seems to play a crucial role in tumorigenesis but also in immunomodulation and immunosensitivity of CRC (Table 2). Microbial dysbiosis and translocation have been correlated with CRC development and progression [6,96]. Intracellular signaling pathways, like unfolded protein response, when activated, fail to reduce the stress of the endoplasmic reticulum, leading to homeostasis disturbances. The activating transcription factor 6 (ATF6) leads to an increase in endoplasmic reticulum capacity and the destruction of defective proteins. Coleman et al. showed that the activated ATF6 leads to intestinal dysbiosis, activation of the immune system and CRC development [96]. It has been demonstrated that the interaction between healthy cells and microbiota or its products could lead to tumorigenesis. *Fusobacterium* spp. are a characteristic example of such a hypothesis. Mice models of sporadic CRC of Apc^Min/+^ were inoculated with *Fusobacterium nucleatum* developed more colon tumors than those inoculated with *Streptococcus* spp. and demonstrated a proinflammatory phenotype without colitis exacerbation [93,97]. Adhesion bacterial proteins FadA and Fap2 seem to be highly involved in tumorigenesis due to *F. nucleatum*. Bacterial FadA promotes tumorigenesis through activation of WNT/β-catenin signaling, whereas Fap2 promotes avoidance of the tumor immunosurveillance. Bacterial Fap2 binds to T cell immunoglobin and the immunoreceptor tyrosine-based inhibition motif domain, an inhibitory receptor expressed by natural killer and T cells [98]. It has been shown that *F. nucleatum* abundance in the gut is associated with dMMR CRC [99]. Lee et al. categorized dMMR CRC tumors into *F. nucleatum*-high and *F. nucleatum*-low tumors. It was revealed that *F. nucleatum*-high tumors are characterized by increased tumor growth and invasion, an immune microenvironment with decreased FoxP3^+^ T cells through the tumor and a high proportion of M2-macrophages in the tumor center. These findings correlate the presence of *F. nucleatum* with pro-cancerous immune responses in dMMR CRC tumors [100]. Other microorganisms of the gut microbiota, such as *Bacteroides* spp. and *Faecalibacterium* sp, are significantly correlated with the abundance of tumor-infiltrating immune cells, which determine, to a significant extent, the immunosurveillance of CRC [13].

Butyrate is a product of bacterial metabolism in the gut and is involved both in the antitumor immune response and in homeostasis. According to the Warburg effect, butyrate leads to a reduction in mRNA expression of cell-cycle regulators and to an increase in mRNA expression of proapoptotic gene Fas, thus leading to apoptosis [101]. However, in dMMR/MSH mice, butyrate leads to hyperproliferation [102], suspends tumor growth and reserves microbial homeostasis by upregulating TLR4 expression and phosphorylation of MAPKs and NF-κΒ in human colon cancer SW480 cells or mouse colon cancer CT26 cells [103]. Modulators of gut microbiota could be a target to improve immunotherapy efficacy in CRC. Indoleamine 2, 3-dioxygenase 1 (IDO1) is a key factor for the resistance of host-microbiome homeostasis. IDO-1 expressed by cancer cells and dendritic cells is a starter enzyme for the tryptophan metabolism pathway and suppresses the immune response against the tumor [15]. IDO-1 interferes in host-microbiome interactions either by the activation of several PRRs, which induces IDO-1 expression, or by bacterial metabolites and their interaction with tryptophan metabolism [15]. TLR4 and TLR9 are the major representatives of PRRs, whereas butyrate and short-chain fatty acids are the main representatives of microbial products in such a process [15,104,105,106,107]. IDO-1 inhibitors, alone or in combination, are under investigation for the treatment of CRC, mostly in the dMMR/MSH setting [14,15]. Phan et al. aimed to assess the therapeutic efficacy of attenuated *Salmonella typhimurium* delivering a small hairpin RNA plasmid targeting IDO (shIDO-ST). Both in vitro and in vivo studies were performed in CT26 and MC38 murine colon cancer models. It was demonstrated that both IDO protein expression and function were reduced and tumor progression was significantly delayed when epacadostat, a known IDO-1 inhibitor, was used [108]. In mouse colon cancer models, anti-CTLA-4 ICI in combination with *Lactobacillus acidophilus* lysates improved antitumor immune response through the increase of infiltrated T cells in the tumor microenvironment. *L. acidophilus* lysates also improved homeostasis in these mice [16]. Moreover, Xu et al., demonstrated that gut microbiota influences the efficacy of anti-PD-1 in MMR-proficient CRC through the glycerolphospholipid metabolic pathway. The authors aimed to evaluate the effects of gut microbiota in CT26 CRC mice treated with different antibiotics on anti-PD-1 response compared to a control group (treated with sterile drinking water). Mice gut microbiota under different antibiotics was remodeled, changing the metabolome and immunity of these mice, thus affecting anti-PD-1 efficacy [17].

Many studies with different regimens are under investigation in order to achieve a wider use of immunotherapy in CRC. These include different ICIs, vaccines, adaptive cell transfer, IDO-1 inhibitors and the carcinoembryonic antigen T cell biphasic antibody [14]. In mouse models under ICIs, it has already been demonstrated that specific bacterial species are linked to better responses. In CRC, the MC38 cell line has been used in two studies that treated the cells with anti-IL-10 plus CpG oligonucleotides and anti-PD-1 or anti-CTLA-4, respectively. It was demonstrated that *Alistipes shahii* and *Ruminococcus* spp. played the role of promoters of antitumor response [109]. Additionally, *Ruthenibacterium lactatiformans*, *Eubacterium limosum*, *F. ulcerans*, *Phascolarctobacterium succinatutens*, *B. uniformis*, *Bacteroides dorei*, *Paraprevotella xylaniphila*, *Parabacteroides johnsonii*, *P. gordonii* and *Alistipes senegalensis* were shown to contribute to improving ICIs efficacy [110].

## 5. Conclusions

Immunotherapy is a new hallmark of solid tumor treatment. Extensions in survival rates and longer-lasting responses have been achieved in various cancers. Melanoma, non-small cell lung cancer, renal cell and urothelial carcinoma are some of them. The rationale of immunotherapy is to boost the immune system response to tumor growth or to overlap the barriers that were created by the tumor itself or the tumor’s microenvironment. Innate and adaptive immunity are equally involved in the inhibition of tumor growth. Numerous efforts have been made through the years, and different molecules have been used aiming to work in concert with the immune system to defeat cancers. Nowadays, ICIs are the main players in this effort. Anti-PD-1, anti-PD-L1 and anti-CTLA-4 are the main immunotherapy options that aid immune system to overcome checkpoints leading to immune escape. However, some cancer types, like CRC, have little or no benefit from such immunotherapy. For all these reasons, several studies are in progress aiming to contribute to better efficacy of immunotherapy.

Gut microbiota plays a crucial role in the interplay of tumor and immune system. A number of studies have shown that different bacterial species can increase the efficacy of immunotherapy [7,24,63,67,109,111]. Gut microbiota interferes with the immune system, mainly through innate immunity. Studies are in progress to evaluate how gut microbiota would enhance prognosis and treatment [42]. Moreover, due to the fact that only dMMR/MSH tumors can benefit from ICIs, new aspects are under investigation for the use of ICIs in MMR-proficient tumors. Therefore, immunotherapy is the new achievement in cancer therapy. The microbiome is the new “human organ” that interferes with all the important functions of the organism. Gut microbiota could be the promoter for a better efficacy of immunotherapy. New aspects to immunotherapy efficacy, even in immunotherapy-resistant cancers, such as MMR-proficient CRC, could be improved by aiming to regulate of the different parts that shape and maintaining the homeostasis of gut microbiota.

## Figures and Tables

**Table 1:** Bacterial species associated with enhancement of immunotherapy in solid tumors.

Tumor	Beneficial Microbiota	Immune Response	Immunotherapy	Reference
Melanoma	*Bifidobacterium* spp.	Increase of dendritic cells function and enhancement of CD8+ T cells priming	anti-PD-L1	[7]
NSCLC, Renal cell or urothelial carcinoma	*A. muciniphila*	CD4+, CD8+ T cells memory towards *A. mucinipilla*	anti-PD-1/anti-PD-L1	[8]
Melanoma	*F. prausnitzii*, *B. thetaiotamicron*, *H. filiformis* and *B. caccae*	CD8+ T cells	anti-PD-1/anti-CTLA-4 or combination	[9]
Melanoma	*F. prausnitzii*, *R. bromii*, *P. pasteri*, *C. hungati* and *P. faecium*	Increased antigen presentation, elevation of CD4+ and CD8+ T cells	anti-PD-1	[10]
Melanoma	*F. prausnitzii* L2-6, *G. formicilitis* ATCC27749, butyrate- producing bacteria SS2-1, *Ruminococcus*, *Lachnospiraceae*, *Clostridium* XIVa and *Blautia* spp.	Low Tregs in peripheral blood, increase in CD4+ cells and serum CD25	anti-CTLA-4	[12]
Melanoma	*Bacteroides* spp.	Activation of Th cells, mobilization of CD11b+ DC to lamina propria	anti-CTLA-4	[26]

**Table 2:** Interference of gut microbiota with immune response in CRC.

Gut Microbiota Component/Agent Influencing Microbiota	Correlation with CRC and/or Immunotherapy	Immune Reaction	Reference
Dysbiotic microbiota due to ATF6 activation	Tumorigenesis	MyD88/TRIF- dependent activation	[96]
*Fusobacterium nucleatum*	tumorigenesis, dMMR CRC	binding to TIGIT on NK and T cells	[93,97,98,99,100]
*Bacteroides* spp. and *Faecalibacterium* sp.	immunosurveillance	Raising of Tregs and M1 TAMs	[13]
Butyrate producing bacteria	dMMR CRC mice models: suspends tumor growth, reserves microbioal homeostasis	upregulation of TLR4, phosphorylation of MAPKs and NF-κΒ	[102,103]
Lysates of *Lactobacillus acidophilus*	improvement of antitumor immune response	Increase of CD8+ Tcells, effector memory T cells, decrease of Tregs and M2 TAMs	[16]
shRNA targeting IDO delivered via attenuated *Salmonella typhimurium*	supresses tumor growth	Increase of intratumoral neutrophil concetration	[108]
Bacteroidales S24-7 in Control group, *A. municiphila* in Vanc group, *Bacteroides* in Coli group	enhace antitumor antiPD-1 activity in MMR-proficient CRC	changes in the expression of INF-γ and IL-2 in tumor microenvironment	[17]
*Alistipes shahii* and *Ruminococcus* spp.	Enhacement of antitumor response of immunotherapy	TLR4, TNF production	[109]
*Ruthenibacterium lactatiformans*, *Eubacterium limosum*, *F. ulcerans*, *Phascolarctobacterium succinatutens*, *Bacteroides uniformis*, *B. dorei*, *Paraprevotella xylaniphila*, *Parabacteroides johnsonii*, *P. gordonii* and *Alistipes senegalensis*	enhancement of antitumor ICI’s effect	Induce interferon-γ+ CD8 T cells	[110]
